# Plasmacytoma as a Mimicker of Colonic Carcinoma in an Elderly Man

**DOI:** 10.1155/2017/4846018

**Published:** 2017-04-19

**Authors:** Sara Mathew George, Eman Ali Aljufairi, Nisha Chandran, Sayed Ali Isa Almahari

**Affiliations:** Department of Pathology, Salmaniya Medical Center, P.O. Box 12, Manama, Bahrain

## Abstract

Multiple myeloma is a neoplastic proliferation of monoclonal plasma cells. Although it is usually restricted to the bone marrow, extraskeletal spread in the form of localised extramedullary collections of malignant plasma cells (plasmacytomas) can occur. However, gastrointestinal tract involvement in multiple myeloma is rare and overt gastrointestinal bleeding from plasmacytoma is uncommon. We report a case of colonic plasmacytoma which presented with bleeding per rectum and was initially misdiagnosed as colonic neuroendocrine carcinoma. Later the patient presented with recurrence of the colonic mass along with multiple lytic bone lesions. The diagnosis of colonic plasmacytoma with progression into multiple myeloma was given. We also discuss here the diagnostic difficulty of plasma cell neoplasms in small biopsies of the colon.

## 1. Introduction

According to the WHO 2016 classification of lymphoid neoplasms, plasma cells tumors are classified into plasma cell myeloma (multiple myeloma), solitary plasmacytoma of bone, and extraosseous plasmacytoma [1]. Multiple myeloma is a clonal malignancy of plasma cells arising from the bone marrow where cells disseminate to form osteolytic bone lesions. It accounts for approximately 10% of all the haematologic malignancies [2]. Although usually restricted to bone marrow, extramedullary involvement in the form of plasmacytoma can occur in up to 20% of cases [3]. Plasmacytomas can occur secondary to multiple myeloma and in such patients they can precede, accompany, or follow the onset of systemic disease [4].

Extramedullary plasmacytomas are rare and vary from 4 to 10% of plasma cell tumors in various studies [5]. Extramedullary plasmacytomas are commonly seen in the upper aerodigestive tract (82%). Only 18% occur in other sites like urinary bladder, central nervous system, thyroid gland, breast, testes, parotid gland, lymph node, and skin. Gastrointestinal tract (GIT) involvement is seen in 7.2–10% of cases [5, 6]. In the GIT, the small intestine is the most commonly involved organ, followed by stomach, colon, and esophagus. Colonic plasmacytomas are uncommon with up to 25–32 cases in the literature [2, 3, 5, 7–12]. In a study conducted at Myeloma Institute of Research & Therapy at Arkansas, GIT involvement was noted in only 24 out of 2584 cases of multiple myeloma. GIT involvement was mostly noted later in the course of the disease [4].

## 2. Case Report

A 65-year-old man with known history of asthma presented to another tertiary hospital with a history of bleeding per rectum for a few days. He underwent a colonoscopy which revealed an irregular thickening with a slightly raised area at the proximal transverse colon. Biopsies taken were reported as neuroendocrine carcinoma. An abdominal CT scan was done at the same hospital and revealed a mass in the proximal part of the transverse colon. No other lesions were seen. No significant lymph nodes or omental deposits were noted.

Patient underwent resection of the transverse colon. The colonic segment showed a disc shaped slightly raised ulcerated mass measuring 23 × 20 mm. An infiltrating high grade malignancy was detected. It was reported to be positive for CD56 and focally positive for NSE and negative for other immunohistochemical stains including cytokeratins (AE1/AE3, CK20, and CK7), synaptophysin, chromogranin A, TTF-1, HMB45, Melan A, S100, and CDX2. The lesion was reported as grade 3 neuroendocrine carcinoma by the primary pathologist.

**Figure 1:**
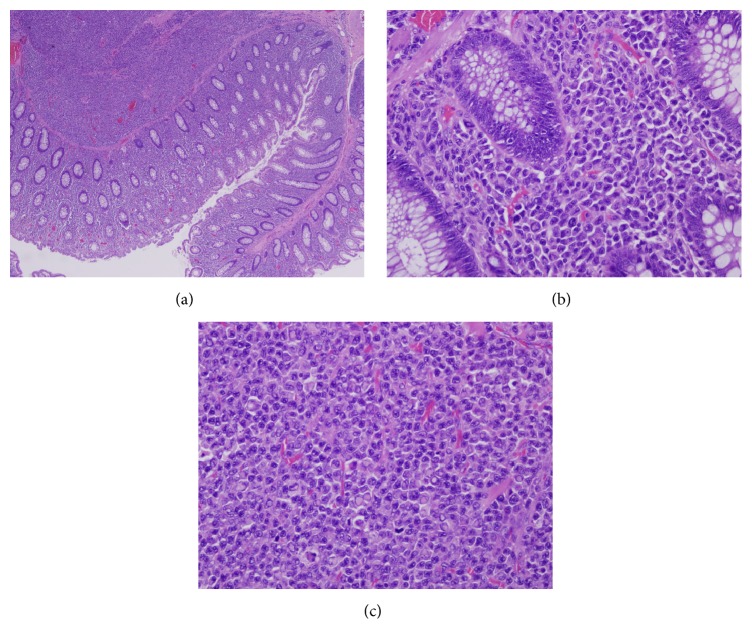
(a) Scanner view shows the tumour cells in the lamina propria and in the submucosa and muscularis propria of the colonic wall (H&E ×40 magnification). (b) High power view shows the morphology of the tumour cells in the lamina propria with preservation of the crypts (H&E ×400 magnification). (c) High power view shows the tumour cells arranged in sheets in the submucosa and muscularis propria. The prominent intranuclear and intracytoplasmic inclusions can be noted (H&E ×400 magnification).

**Figure 2:**
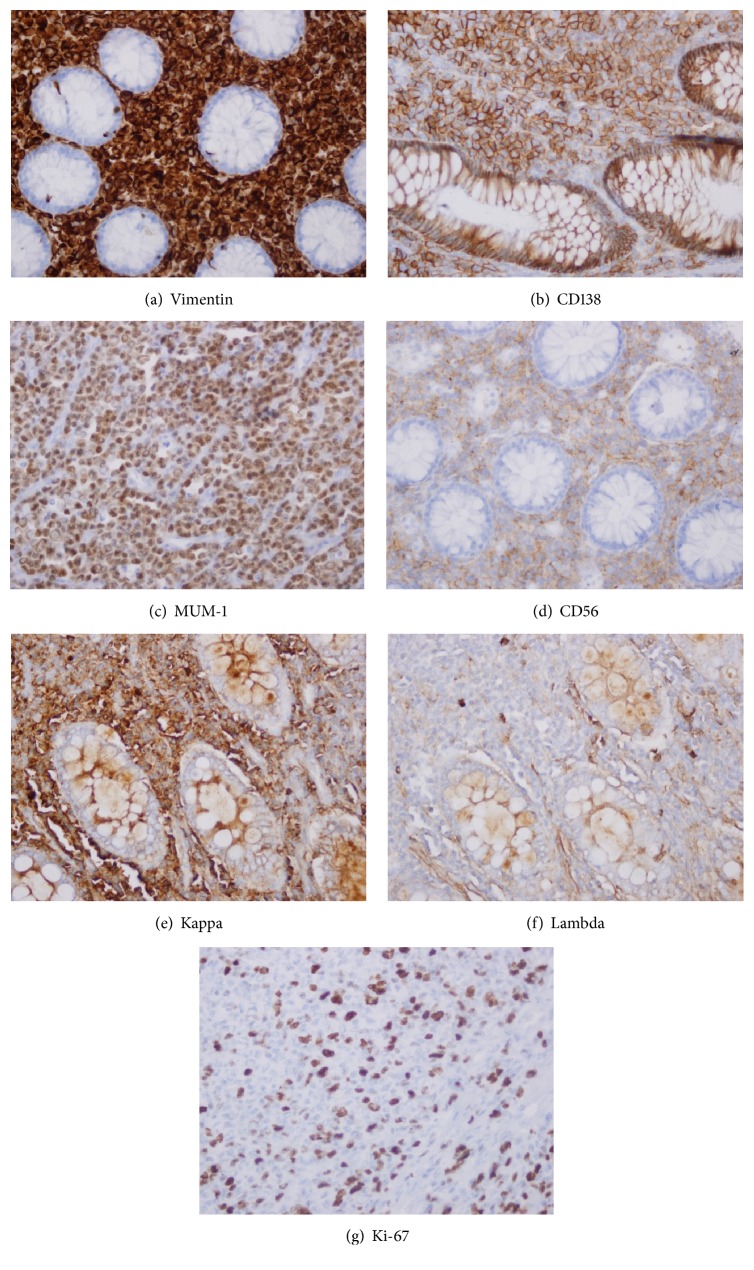
(a) High power view shows the tumour cells staining positive for vimentin (IH stain with haematoxylin counterstain ×400 magnification). (b) High power view shows tumour cells are positive for CD138 (IH stain with haematoxylin counterstain ×400 magnification). (c) High power view shows that tumour cells are positive for MUM 1 (IH stain with haematoxylin counterstain ×400 magnification). (d) High power view shows that tumour cells are positive for CD56 (IH stain with haematoxylin counterstain ×400 magnification). (e) High power view shows that tumour cells are positive for kappa light chain (IH stain with haematoxylin counterstain ×400 magnification). (f) High power view shows that tumour cells are negative for Lambda light chain (IH stain with haematoxylin counterstain ×400 magnification). (g) High power view shows the ki67 proliferation index in the tumour (IH stain with haematoxylin counterstain ×400 magnification).

The patient was referred to the oncology centre in our hospital for further management. He had a whole body scan which showed no lesions anywhere else and was advised with follow-up. After a period of 9 months, he developed right shoulder pain and bleeding per rectum again. A whole body CT scan showed multiple osteolytic lesions involving rib, clavicle, and bilateral femoral shafts. A 11 × 10 cm mass was also noted in the retroperitoneum posterior to head of pancreas, attached to posterior wall of stomach and involving the duodenum. It was diagnosed as a recurrence of the initial colonic mass. Colonoscopy was done and showed a raised lesion at the anastomotic site from which biopsies were taken. The colonic biopsies showed small to medium sized, oval to round neoplastic cells infiltrating the lamina propria without crypt destruction. The cells had round nuclei and prominent nucleoli. No grouping or glandular pattern was noted. Immunohistochemical stains were done on this biopsy and showed negative staining for cytokeratins (AE1/AE3, CK8/18), chromogranin A, NSE, and synaptophysin. As the tissue in the small biopsy was exhausted without confirmation of the original diagnosis of neuroendocrine carcinoma, we requested for the original blocks of the tumor from the primary hospital. We reviewed the slides, which showed a malignant neoplasm similar to that seen in the biopsy involving all layers of the colonic wall. These tumour cells were seen in the lamina propria in between the preserved crypts and infiltrating the muscularis propria and beyond, but confined to the bowel wall (Figure 1(a)). The tumour cells had plasmacytoid morphology with marked pleomorphism and many intranuclear and intracytoplasmic inclusions (Figures 1(b) and 1(c)). Additional immunohistochemical stains were performed. The tumor cells were negative for the cytokeratins, lymphoid markers, and all mesenchymal markers except vimentin (Figure 2(a)). This immunohistochemical reaction pattern along with the plasmacytoid appearance of the tumor cells led us to think about plasma cell neoplasm. The plasma cell markers CD138, MUM-1, and CD56 were positive along with positivity for kappa light chain and negativity for Lambda light chain staining (Figures 2(b)–2(f)). Ki-67 proliferation index was 30% (Figure 2(g)).

In correlation with the multiple lytic lesions in the bone and with the above morphologic and immunohistochemical findings in the colonic mass, we made a diagnosis of multiple myeloma. Serum electrophoresis showed a monoclonal band of beta globulins. Immunofixation electrophoresis depicted IgA kappa type. Bone marrow trephine biopsy revealed trilineage haematopoiesis with increased plasma cells.

Patient was then referred to the hematooncology department for further management of multiple myeloma, but he expired before proper treatment could be started.

## 3. Discussion

Plasma cell neoplasms are more common in men in the 4th to 7th decades [11] and similar distribution is noted in plasmacytomas of the colon [10], as in the present case.

The presenting symptom of GIT plasmacytomas depends upon the site and extent of involvement. Intestinal obstruction, bleeding per rectum, and weight loss may be the presenting symptoms in colonic or small bowel involvement [3]. In our case, patient presented with per rectal bleeding and a colonic mass was detected on further investigation. Clinical presentation can mimic carcinoma, intestinal tuberculosis, or inflammatory bowel disease [10, 12].

Endoscopically, it may present as ulcers, masses, polyps, plaques, or diffuse infiltrating lesion and can mimic other conditions like neoplasms (carcinoma, lymphoma) or amyloidosis. Pathological and immunohistochemical studies are necessary for making a correct diagnosis [3, 7].

Usually multiple myeloma involving the GIT shows more plasmablastic morphology than usual multiple myeloma [4]. Our case showed pleomorphic cells with intracytoplasmic and intranuclear inclusions.

Because of the rarity of the GIT plasmacytoma or its involvement by multiple myeloma, it is rarely considered in the differential diagnosis of colonic mass during a histopathology workup [5, 13].

Situation is more difficult when there is no previous history of multiple myeloma and it is a challenge to the histopathologist when the characteristic plasma cell morphology is not evident with many pleomorphic cells as in our case. These may be causes of misinterpretation of this case as poorly differentiated carcinoma or neuroendocrine carcinoma.

We present this unusual case to highlight the importance of considering this entity in the differential diagnosis of an undifferentiated malignant neoplasm of the GIT and selection of a proper immunohistochemical panel to arrive at the diagnosis.

The differential diagnoses considered should include malignant lymphoma, poorly differentiated adenocarcinoma, signet ring cell adenocarcinoma, neuroendocrine carcinoma, high grade sarcoma, and extramedullary plasmacytoma [9, 13]. An immunohistochemical stain panel is required for definite diagnosis. It should include wide spectrum cytokeratins, hematolymphoid malignancy markers, and mesenchymal differentiation markers. As in our case the absence of wide range of cytokeratins (AE1/AE3, CK8/18, and CK7, CK 20 along with CDX2) excludes poorly differentiated colonic carcinomas including the plasmacytoid carcinoma. Chromogranin A, synaptophysin, and NSE being negative exclude neuroendocrine carcinoma. Despite vimentin positivity, the absence of all other specific mesenchymal differentiation markers should lead to considering other differential diagnoses before calling it undifferentiated sarcoma NOS. LCA (Leukocyte common antigen) and other lymphoid markers (CD3, CD20, CD7, CD4, CD79a, CD30, CD68, PAX5, ALK1, and TdT) negativity exclude malignant lymphoma. Negative MPO, CD34, and CD68 rule out myeloid neoplasms. S-100, HMB45, and MelanA, being negative, exclude melanoma. The positive reaction of vimentin, CD138, MUM-1, and CD56 strongly supports plasma cell origin along with monoclonal kappa light chain positivity [13].

In the gastrointestinal tract, the diagnosis of plasmacytoma or myeloma with extramedullary involvement should be made after excluding the more common involvement of non-Hodgkin lymphoma with plasmacytic differentiation specially extranodal marginal zone lymphoma of mucosa associated lymphoid tissues (MALT lymphoma) and lymphoplasmacytic lymphoma (LPL) in cases with bland appearance and plasmablastic lymphoma in case of anaplastic myeloma. MALT lymphoma and LPL usually show a mixture of CD20 positive lymphocytes and CD138 positive plasma cells, while myeloma shows only plasma cells. The lytic lesions seen in multiple myeloma are also not seen in MALT and LPL cases. Multiple myeloma can also be associated with t(4;14) or t(14;16), or t(14;20) (detected by FISH) and/or aberrant expression of cyclinD1 or CD56 (detected by immunohistochemical staining), which is not seen in MALToma or LPL. Plasmablastic lymphoma usually is associated with immunodeficient state and positive for EBV, while multiple myeloma is not [14].

Prognosis of solitary extramedullary plasmacytoma is good with median survival of 9.5 years and 56% of patients were free of systemic disease after 5 years [15]. But prognosis of multiple myeloma with GIT involvement is poor, as it is associated with adverse biological features. Even after aggressive treatment with chemotherapy and stem cell transplantation in systemic diseases, the median survival after diagnosis of GIT involvement was 7 months [4].

## 4. Conclusion

It is necessary to give a correct histopathological diagnosis that will determine the right treatment. Although GIT plasma cell neoplasms are rare, they must be included in the differential diagnosis of patients with GIT bleeding, especially in those with a history of multiple myeloma. Pathologists and radiologists should be aware of this entity.
